# Food and Brain Work

**Published:** 1885-03

**Authors:** 


### FOOD AND BRAIN WORK.

An organism which is doing brain work as well as muscular work requires higher and better food than an organism in which the brain is comparatively idle and only the lower centres and the muscles do much work. Undoubtedly the effort of brain work is to strengthen the brain and to render it less likely to become abnormal in its structure or disorderly in its activity than if it were idle. Such exercise as the brain receives in education, properly so-called—that is, development of the faculties—stimulates nutrition, and in so doing increases the need of food. Excessive activity with anxiety is not good at all, and ought to have no place in the educational process. Worry is fatal to good work, and to worry the growing brain of a child with work, is to maim and cripple its organization, doing irreparable, because structural, mischief, the effect of which must be life-long. "Tension" in work is not a proof of strength, but of weakness. A well developed and healthy grown brain works without tension of any kind. The knit brow, straining eyes, and fixed attention of the scholar are not tokens of power, but of effort. The true athlete does not strain and pant when he puts forth his strength. The intellectual man with a strong mind does his brain work easily. Tension is friction, and the moment the toil of a *growing* brain becomes laborious it should cease. We are, unfortunately, so accustomed to see brain work done with effort that we have come to associate work with effort, and to regard "tension" as something tolerable, if not natural. As a matter of fact no man should knit his brow as he thinks or in any way evince effort as he works. The best brain work is done easily, with a calm spirit, an equable temper, and in jaunty mood, all else is the toil of a weak or ill-developed brain straining to accomplish a task which is relatively too great for it—*Lancet.*

